# Dysregulation of miRNAs in Soft Tissue Sarcomas

**DOI:** 10.3390/cells13221853

**Published:** 2024-11-08

**Authors:** Stefano Zoroddu, Angela Lucariello, Antonio De Luca, Luigi Bagella

**Affiliations:** 1 Department of Biomedical Sciences, University of Sassari, Viale San Pietro 43/b, 07100 Sassari, Italy; 2 Department of Sport Sciences and Wellness, University of Naples “Parthenope”, 80100 Naples, Italy; 3 Department of Mental and Physical Health and Preventive Medicine, Section of Human Anatomy, University of Campania “Luigi Vanvitelli”, Via Costantinopoli 16, 80138 Naples, Italy; 4 Sbarro Institute for Cancer Research and Molecular Medicine, Centre for Biotechnology, College of Science and Technology, Temple University, Philadelphia, PA 19122, USA

**Keywords:** miRNA, sarcoma, soft tissue sarcoma, rhabdomyosarcoma, tumorigenesis, biomarkers

## Abstract

MicroRNAs (miRNAs) are pivotal regulators of gene expression, influencing key cellular processes such as proliferation, differentiation, apoptosis, and metastasis. In the realm of sarcomas—a diverse group of malignant tumors affecting soft tissues and bone sarcomas—miRNAs have emerged as crucial players in tumorigenesis and tumor progression. This review delves into the intricate roles of miRNAs across various soft tissue sarcoma subtypes, including rhabdomyosarcoma, liposarcoma, leiomyosarcoma, synovial sarcoma, fibrosarcoma, angiosarcoma, undifferentiated pleomorphic sarcoma (UPS), and malignant peripheral nerve sheath tumor (MPNST). We explore how dysregulated miRNAs function as oncogenes or tumor suppressors, modulating critical pathways that define the aggressive nature of these cancers. Furthermore, we discuss the diagnostic and prognostic potential of specific miRNAs and highlight their promise as therapeutic targets. By understanding the miRNA-mediated regulatory networks, this review aims to provide a comprehensive overview of current research while pointing towards future directions for miRNA-based therapies. Our findings underscore the potential of miRNAs to transform the landscape of sarcoma treatment, offering hope for more precise, personalized, and effective therapeutic strategies.

## 1. Introduction 

Sarcomas are a heterogeneous group of malignant tumors originating in the mesenchyme. They are classified into bone sarcomas and soft tissue sarcomas (STSs) which may affect fibrous, cartilaginous, muscular, synovial, and adipose tissue. Although mesenchymal tissues constitute the majority of body mass, they are rather rare tumors, representing about 1% of human malignancies [1,2]. This rarity, combined with their significant histological diversity and varying clinical behaviors, makes sarcomas a complex group of cancers to diagnose and treat. Moreover, the molecular mechanisms underlying sarcoma development and progression are not fully understood, necessitating further research to uncover the genetic and epigenetic factors involved. Improved understanding of these mechanisms could lead to better diagnostic tools, prognostic markers, and more targeted therapeutic strategies, ultimately enhancing patient outcomes in this challenging group of malignancies.

**Figure 1:**
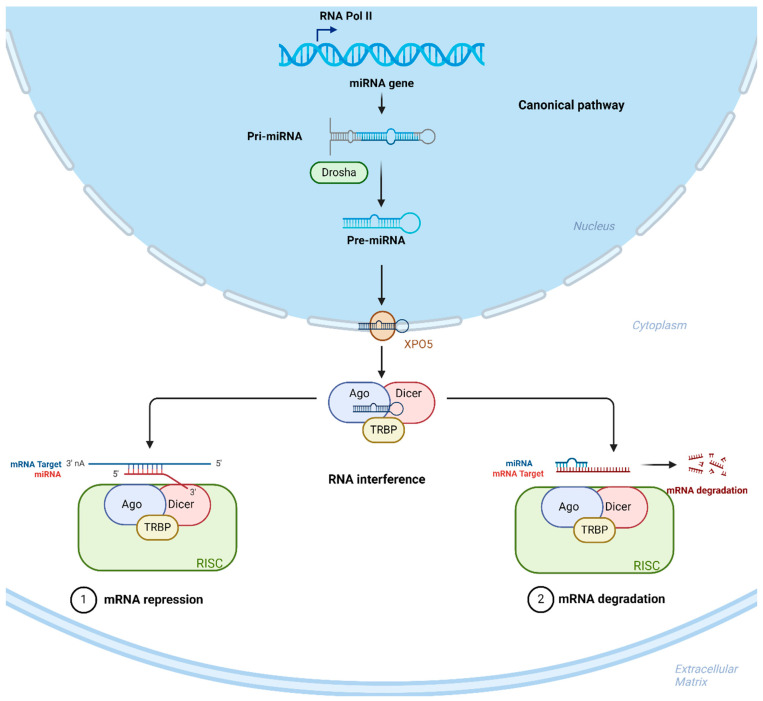
Biogenesis and function of miRNAs. The miRNAs are initially translated into pre-miRNA sequences, which are subsequently transformed into mature miRNAs and transported into the cytoplasm, where they attach to the target mRNA to be silenced or degraded.

MicroRNAs (miRNAs) are a class of non-coding RNAs that are involved in several regulatory process, and their functions are essential for the maintenance of physiological conditions [3]. They are involved in the balance of several physiological and pathophysiological processes, including proliferation, differentiation and cell death [4]. They fulfil their function by negatively regulating the expression of target miRNAs at the post-transcriptional level through base pairing by preventing translation or directly destroying the targets [5]. The biogenesis of miRNAs occurs through several sequential steps (Figure 1). Initially, miRNAs are transcribed in the nucleus as primary transcripts (pri-miRNAs), which can be several kilobases long and contain one or more stem–loop structures. These pri-miRNAs are recognized and cleaved by the microprocessor complex, which includes the RNase III enzyme DROSHA and its cofactor DGCR8, resulting in the formation of a shorter, hairpin-shaped precursor miRNA (pre-miRNA) [6]. This pre-miRNA is then transported from the nucleus to the cytoplasm by exportin-5 (XPO5) in a GTP-dependent manner [7]. Once in the cytoplasm, the pre-miRNA is further processed by the RNase III enzyme DICER, which cleaves the hairpin to produce a small double-stranded RNA of approximately 22 nucleotides. One strand of this duplex, known as the guide strand, is then incorporated into the RNA-induced silencing complex (RISC), where it is bound by an argonaute (AGO) protein [8]. The RISC with the guide miRNA is now capable of binding to complementary sequences in target mRNAs, leading to translational repression or mRNA degradation, thereby regulating gene expression post-transcriptionally.

The development of cancer can also be affected by dysregulation of these molecules. Depending on the role and function of their targets, miRNAs may behave as onco-suppressors or tumor suppressors [9]. In rhabdomyosarcoma (RMS), some miRNAs are deregulated in comparison to the physiological skeletal muscle [10]. Indeed, they can regulate the process of skeletal myogenesis by monitoring the proliferation and differentiation of myoblasts. The efficiency of these finely regulated mechanisms can cause uncontrolled cell proliferation leading to neoplasia. This review article analyzes deregulated miRNA expression in various types of sarcomas, including a detailed discussion of RMS and other significant subtypes. In addition to highlighting the specific miRNAs involved in these neoplasms, we explore the key mechanisms underlying the upregulation or downregulation of miRNA expression in sarcomas. This review aims to provide insights into the role of miRNAs in the pathogenesis of sarcomas, highlighting their potential as therapeutic targets and future directions for treatment strategies.

## 2. miRNA Dysregulation in STS 

The involvement of miRNAs in sarcoma biology is extensive and varies across different histological subtypes. In STSs, studies have identified several miRNAs with altered expression profiles that are involved in key regulatory pathways (Table 1).

### 2.1. Rhabdomyosarcoma

RMS is the most common form of STS in pediatric patients. It has an incidence of 4.5 cases for 1 million children with a 5-yearsurvival rate of about 70% with standard therapy such as chemotherapy, radiotherapy and surgery. The main cause of death remains metastasis, drug resistance, or tumor recurrence [53]. Recent findings highlight the involvement of epigenetic regulators like enhancer of zeste homolog 2 (EZH2), which has been linked to disrupted skeletal muscle differentiation and tumorigenesis in rhabdomyosarcoma, suggesting its role as a key mediator in RMS progression and as a potential therapeutic target [54]. Given the role of EZH2 in modulating myogenic differentiation and promoting carcinogenesis, targeting EZH2 has emerged as a promising strategy in RMS treatment and in broader cancer therapy, potentially offering a new avenue to combat drug resistance and tumor recurrence [55]. 

According to the World Health Organization (WHO) Classification of Tumours (5th edition, Soft Tissue and Bone, 2020) [56], RMS is categorized into four distinct subtypes based on clinicopathological and molecular genetic features: embryonal (eRMS), alveolar (aRMS), spindle cell/sclerosing, and pleomorphic RMS. The spindle cell/sclerosing subtype has been found to harbor mutations in genes such as MYOD1, VGLL2, and gene fusions like NCOA2-associated and RAB3IP-HMGA2 [57,58]. eRMS is clinically less severe than aRMS, despite constituting 60 % of all cases of RMS. Almost all aRMS are related to a reciprocal translocation of chromosomes t (2;13) and t (1;13) involving the genes PAX3 and PAX7 reciprocally and the transcription factor FOXO1 [53]. These fusion oncoproteins, in particular PAX3-FOXO1, are linked to disease aggressiveness, tumor invasion, and metastasis [59]. Indeed, aRMS tumors are defined as PAX-fusion-positive and are associated with more aggressive phenotype and poorer prognosis than PAX-fusion-negative eRMS tumors. A better classification of RMS tumors is based on fusion status rather than histology, dividing them into fusion-positive RMS (FP-) or fusion-negative RMS (FN-), as the absence of a fusion oncoprotein in some RMS cases makes them indistinguishable from eRMS in terms of prognosis and overall survival [60]. eRMS, which has a better prognosis, is variable in terms of oncogenic mutations and may include loss of imprinting (LOI) on chromosome 11, mutations in the IGF-2, p16 and p53 genes, or epigenetic abnormalities such as long intergenic non-coding RNAs (lncRNAs) like H19.

Given the limitations of conventional therapies, novel treatment strategies are being explored to target specific molecular mechanisms associated with RMS. Recent studies have shown that verteporfin, a photosensitizing agent, exhibits anti-proliferative effects in both embryonal and alveolar RMS cell lines, highlighting its potential application as an adjunctive therapy to curb aggressive tumor growth [61]. The bromodomain inhibitor JQ1 has provided novel insights into RMS treatment by targeting transcriptional programs critical for tumor survival, thereby opening up new perspectives for therapeutic interventions in RMS management [62]. Combining 12-O-tetradecanoylphorbol-13-acetate with EZH2 inhibition has shown promise in promoting myogenic differentiation in embryonal RMS cells, suggesting a potential therapeutic approach to restore normal differentiation pathways and mitigate RMS malignancy [63].

Pediatric and adult RMS exhibit notable differences in terms of natural history, clinical behavior, and outcomes. Pediatric RMS more commonly affects the head, neck, and genitourinary tract, whereas adult RMS shows a preference for the extremities. The prognosis is also significantly different, with a 5-year overall survival (OS) in adults ranging from approximately 20% to 40%, which is much poorer compared with pediatric cases [64,65]. aRMS and eRMS subtypes are characterized by the loss of myoblastic differentiation caused by various genetic and epigenetic factors. The myogenic process is coordinated by a complex regulatory network of gene expression exerted by myogenic regulatory factors (MRFs) and certain transcription factors that enable specific transcription of muscle genes [53]. Indeed, RMS has several features that could link it to normal muscles, including, among others, a high expression of proteins belonging to growth-promoting MRFs, such as MyoD, myogenin, desmin or muscle-specific miRNAs (miRs-1, -133b or -206). Despite the expression of several mediators, including MyoD, a key regulator of myogenesis, RMS continues to exist at various stages of myogenic differentiation. This abnormal pattern of cell maturation is accompanied by a series of epigenetic changes, ranging from altered methylation and downregulation of methyltransferase expression to disturbed expression of lncRNA or miRNA [12,66]. Therefore, it might be a good strategy to identify new therapeutic targets that could be used in the clinical field. In recent years, miRNAs have assumed a very important role in the control of abnormal myogenesis. These mediators, together with other epigenetic factors and classical myogenic transcription factors, appear to be significantly more crucial for the onset of RMS. These miRNAs can be broadly categorized into muscle-specific miRNAs (myomiRs), which are primarily involved in muscle cell regulation, and non-muscle-specific miRNAs that affect various cellular pathways contributing to cancer development.

### 2.2. Muscle-Specific miRNAs in RMS

Muscle-specific miRNAs, including miR-1, miR-133a/b, and miR-206, are deeply involved in the regulation of myogenesis and are frequently dysregulated in RMS [67,68]. These miRNAs play critical roles in promoting muscle differentiation, and their altered expression in RMS is associated with failure to achieve full differentiation, a characteristic feature of RMS cells.

miR-1 and miR-206 have been extensively studied in the context of RMS [12,67,69]. Research by Yan et al. demonstrated that these miRNAs are significantly downregulated in both embryonal and alveolar RMS subtypes [68]. Their study showed that miR-1 and miR-206 promote myogenic differentiation by targeting genes involved in maintaining an undifferentiated, proliferative state, such as PAX3 and c-MET [68]. In eRMS, where PAX3 is not fused with FOXO1, the restoration of miR-1 or miR-206 can lead to the downregulation of PAX3, resulting in reduced proliferation and enhanced differentiation. However, in aRMS, characterized by the PAX3-FOXO1 fusion gene, these miRNAs are unable to effectively suppress the fusion oncoprotein, highlighting a subtype-specific difference in miRNA functionality [68].

Similarly, miR-133a and miR-133b are downregulated in RMS, as shown by Rao et al. [11]. These miRNAs regulate muscle cell proliferation and differentiation by targeting serum response factor (SRF), which controls muscle-specific gene expression. The downregulation of miR-133a/b in RMS promotes an undifferentiated state, facilitating tumor growth. Rao and colleagues found that restoring miR-133a/b expression in RMS cells resulted in a significant decrease in cell proliferation and induced differentiation, underscoring their potential as therapeutic targets [11].

Additionally, the miR-29 family (miR-29a, miR-29b, and miR-29c) has been identified as a critical player in muscle differentiation and RMS pathology. Wang et al. demonstrated that miR-29 is downregulated in RMS, particularly in aRMS, and acts as a tumor suppressor by targeting genes involved in extracellular matrix remodeling and cell adhesion, such as COL1A1, COL1A2, and MMP2 [14]. Their study showed that miR-29 downregulation correlates with increased cell migration and invasion, as well as resistance to differentiation, positioning it as a key regulatory element in RMS development [14].

### 2.3. Non-Muscle-Specific miRNAs in RMS

In addition to muscle-specific miRNAs, several non-muscle-specific miRNAs are dysregulated in RMS and contribute to its malignant behavior by influencing various cellular pathways.

miR-183 is one such miRNA identified as an onco-miR in RMS [16,70]. Sarver et al. found that miR-183 is upregulated in both eRMS and aRMS, where it promotes cell migration and invasion by targeting the tumor suppressor genes early growth response 1 (EGR1) and phosphatase and tensin homolog (PTEN) [15]. The loss of EGR1 and PTEN due to miR-183 upregulation contributes to increased tumor aggressiveness and a poorer prognosis [15].

Another important non-muscle-specific miRNA is miR-9, which has been implicated in the metastatic potential of aRMS. Armeanu-Ebinger et al. [17] reported that miR-9 is upregulated in aRMS and correlates with enhanced metastatic ability by targeting E-cadherin, a key molecule in maintaining cell–cell adhesion [17]. The downregulation of E-cadherin by miR-9 facilitates cell detachment and migration, prom oting metastasis [17].

miR-214 also plays a significant role in RMS [18]. While it is not muscle-specific, it is involved in promoting myogenic differentiation and inhibiting tumor growth. Huang et al. demonstrated that miR-214 is downregulated in RMS and that its re-expression induces differentiation by targeting N-Ras, a gene involved in cell proliferation and survival [18]. Their study showed that reducing N-Ras levels through miR-214 overexpression decreased RMS cell growth and promoted differentiation, highlighting its potential therapeutic value [18].

miR-203 acts as a tumor suppressor in RMS, targeting p63 and leukemia inhibitory factor (LIF), both of which are involved in maintaining an undifferentiated state [19]. Diao et al. showed that downregulation of miR-203 is common in RMS and that its reintroduction promotes terminal myogenic differentiation and inhibits RMS cell proliferation and migration [19]. This suggests that miR-203 could be a valuable target for therapeutic strategies aimed at promoting differentiation and reducing tumor aggressiveness [19].

Another tumor-suppressive miRNA, miR-28-3p, has been identified in RMS, and its significant role in controlling metastasis has been established. It functions by targeting EZR (ezrin), a protein that is critical for cell adhesion and migration. There is evidence that overexpression of EZR is linked to increased invasiveness in RMS. The overexpression of miR-28-3p in RMS cell lines has been demonstrated to result in a reduction of EZR levels, which in turn impairs cell adhesion to endothelial cells and diminishes migration capabilities. These effects identify miR-28-3p as a potential therapeutic target for the mitigation of RMS invasiveness and metastatic spread [22,71,72,73].

miR-27a has also been identified as a tumor-suppressive miRNA with key roles in RMS. It directly targets the oncogenic fusion protein PAX3-FOXO1, which is highly expressed in alveolar RMS and contributes to its aggressive phenotype. By downregulating PAX3-FOXO1, miR-27a can impede cell proliferation and invasiveness in RMS. Moreover, it is postulated that miR-27a interacts with HDAC3-SMARCA4, a regulatory pathway implicated in myogenic differentiation, which further underscores its potential utility as a therapeutic agent in RMS [23].

miR-181a/212, known as a pro-myogenic “miRNA cocktail”, has also been shown to inhibit RMS progression by promoting differentiation. This miRNA pair markedly enhances myoblast fusion, augmenting the myotube formation index in RMS cells and thereby facilitating a transition towards a more differentiated, less proliferative state. Furthermore, miR-181a/212 has been demonstrated to impede the migration and proliferation of RMS cells, offering a promising avenue for pro-differentiation therapy to counteract the aggressive behavior associated with RMS [24].

miR-450b-5p is another miRNA involved in RMS pathogenesis. Sun et al. found that miR-450b-5p is tightly regulated by TGF-β1 and contributes to RMS growth and differentiation [20]. By targeting components of the TGF-β signaling pathway, miR-450b-5p influences the balance between RMS cell proliferation and differentiation, suggesting that targeting this miRNA could offer new therapeutic opportunities [20].

Finally, miR-22 and miR-378a-3p have also been implicated in RMS. Bersani et al. demonstrated that miR-22 is downregulated in RMS and that its restoration can block tumor growth and dissemination by regulating genes associated with cell cycle control and differentiation [21]. Meanwhile, Gasparini et al. showed that miR-378a-3p modulates RMS cell apoptosis, migration, and viability through the downregulation of insulin-like growth factor 1 receptor (IGF1R), further suggesting that these miRNAs may serve as therapeutic targets in RMS [21,74].

The diverse roles of both muscle-specific and non-muscle-specific miRNAs in RMS development highlight the complexity of this disease and its regulatory networks. While myomiRs such as miR-1, miR-133, and miR-206 are crucial for maintaining muscle cell differentiation and are downregulated in RMS, non-muscle-specific miRNAs like miR-183, miR-9, and miR-214 further contribute to RMS progression by modulating key oncogenic pathways. Understanding the specific contributions and interactions of these miRNAs could lead to more targeted and effective therapeutic approaches for RMS, particularly by reversing the dysregulated miRNA profiles that promote tumor growth and metastasis.

### 2.4. miRNAs in Liposarcoma

Liposarcomas are a heterogeneous group of malignant tumors that originate from adipocytic precursor cells and represent approximately 15% of all STSs [75,76]. They are classified into several histological subtypes, including well-differentiated (WDLSs), dedifferentiated (DDLSs), myxoid/round cell (MLSs), and pleomorphic liposarcomas (PLSs), each with distinct molecular and clinical characteristics [77]. Investigations into liposarcomas have revealed a few miRNAs whose dysregulation drives key tumorigenic processes. Whether acting to promote or inhibit tumor growth, these miRNAs represent potential molecular targets for novel treatment strategies.

### 2.5. Tumor Suppressor miRNAs in Liposarcoma

miR-143 and miR-145 are among the most extensively studied tumor-suppressive miRNAs in liposarcomas [78]. Both miRNAs are significantly downregulated in WDLS and DDLS. Ugras et al. [78] reported that miR-143 directly targets several oncogenes and proteins involved in cell cycle regulation, such as B-cell lymphoma 2 (BCL2), topoisomerase 2A, protein regulator of cytokinesis 1 (PRC1), and polo-like kinase 1 (PLK1) [27,78]. These proteins are crucial for cell proliferation and survival. Downregulation of miR-143 leads to increased expression of these proteins, contributing to tumor growth and progression. In contrast, re-expression of miR-143 in DDLS cell lines significantly inhibits cell proliferation and induces apoptosis, demonstrating its potential as a therapeutic target in liposarcoma treatment.

Similarly, miR-145, often co-expressed with miR-143, has been shown to suppress liposarcoma progression by targeting genes involved in maintaining the stemness and undifferentiated state of cancer cells [28]. Gits et al. reportedthat miR-145 targets transcription factors like OCT4, SOX2, and KLF4, which are essential for the plasticity and proliferation of cancer cells [28]. The reintroduction of miR-145 in liposarcoma models resulted in reduced tumor growth and induced differentiation, highlighting its critical role in tumor suppression.

miR-451 is another tumor-suppressive miRNA that has been identified in liposarcomas. Its expression is significantly reduced in liposarcoma tissues compared with benign adipocytic tumors [28]. Gits et al. demonstrated that the overexpression of miR-451 in liposarcoma cells leads to decreased cell proliferation, impaired cell cycle progression, and induced apoptosis [28]. These effects are mediated through the direct targeting of 14-3-3zeta, a protein involved in cell cycle regulation and apoptosis inhibition, further suggesting that miR-451 could serve as a valuable therapeutic target.

### 2.6. Oncogenic miRNAs in Liposarcoma

On the other hand, several miRNAs function as oncogenes in liposarcomas, promoting tumor growth and metastasis. miR-155 is significantly overexpressed in myxoid/round cell, dedifferentiated, and pleomorphic liposarcomas compared with normal adipose tissue [26]. Zhang et al. found that miR-155 promotes tumorigenesis by targeting casein kinase 1 alpha (CK1α), leading to enhanced β-catenin signaling and increased cyclin D1 expression [30]. This upregulation drives cell proliferation and tumor growth. Inhibition of miR-155 in liposarcoma cell lines reduced tumor growth in vitro and in vivo, highlighting its potential as a target for therapeutic intervention [30].

miR-26a-2 is another oncogenic miRNA that is overexpressed in WDLS and DDLS [40,47]. Located near the MDM2 gene, miR-26a-2 promotes liposarcoma growth by targeting the homeobox protein A5 (HOXA5) and regulator of chromosome condensation and BTB domain-containing protein 1 (RCBTB1). Lee et al. demonstrated that the overexpression of miR-26a-2 enhances cellular proliferation, survival, and invasion, contributing to an aggressive tumor phenotype [31]. Targeting miR-26a-2 or restoring its downstream targets could provide new therapeutic opportunities for managing liposarcoma.

In myxoid liposarcoma, characterized by the t (12;16) (q13;p11) translocation, miR-486 is significantly downregulated [26,32,79]. Borjigin et al. reported that miR-486 interacts with plasminogen activator inhibitor-1 (PAI-1), a key promoter of cellular proliferation and invasion [32]. Downregulation of miR-486 leads to increased PAI-1 expression, promoting tumor growth and metastasis. Restoring miR-486 expression reduces tumor cell growth and invasiveness, suggesting it as a potential therapeutic target for myxoid liposarcoma.

The intricate involvement of miRNAs in liposarcoma highlights their crucial influence on tumor behavior, playing both protective and harmful roles. Tumor-suppressive miRNAs such as miR-143, miR-145, miR-451, and the let-7 family contribute to halting cancer growth by limiting proliferation and triggering apoptosis. In contrast, oncogenic miRNAs like miR-155 and miR-26a-2 drive tumor progression by fostering cell survival and increasing malignancy. Gaining deeper insights into how these miRNAs orchestrate cellular processes can pave the way for innovative diagnostic tools and therapeutic approaches. Tailoring miRNA-based treatments to the specific molecular characteristics of liposarcoma subtypes holds promise for more effective interventions. Continued research is essential to bring these miRNA-focused strategies into clinical practice and improve outcomes for patients.

### 2.7. miRNAs in Leiomyosarcoma

Leiomyosarcoma (LMS) is a malignant smooth muscle tumor that accounts for approximately 10% of all STSs [80]. It can arise at various body sites, including the uterus, retroperitoneum, and extremities. LMS is characterized by its aggressive clinical behavior, often leading to local recurrence and metastasis [81]. The molecular mechanisms underlying LMS pathogenesis are complex, involving both genetic and epigenetic alterations. Recent findings emphasize the significant influence of miRNAs in LMS, where they regulate gene expression and play essential roles in tumor progression, metastasis, and treatment outcomes.

### 2.8. Tumor Suppressor miRNAs in Leiomyosarcoma

Several miRNAs have been identified as tumor suppressors in LMS, playing significant roles in inhibiting cell proliferation, promoting apoptosis, and suppressing metastasis.

miR-152 is a notable tumor suppressor miRNA that is downregulated in LMS [33]. This miRNA has been shown to target the tyrosine-protein kinases MET and KIT, both of which are crucial for cell growth and survival [33]. MET, when activated by hepatocyte growth factor (HGF), promotes cellular invasion and migration, while KIT is involved in cell proliferation via downstream signaling pathways such as PI3K/AKT. Pazzaglia et al. demonstrated that downregulation of miR-152 in LMS is associated with increased levels of MET and KIT, enhancing tumor aggressiveness [33]. Experimental upregulation of miR-152 in LMS cell lines reduces MET and KIT levels, diminishes PI3K/AKT pathway activity, induces S-phase cell cycle arrest, and enhances apoptotic cell death, highlighting its therapeutic potential [27].

The let-7 family, including let-7b and let-7g, is also downregulated in LMS. These miRNAs are known to target HMGA2, an oncogene frequently overexpressed in LMS and associated with increased cell proliferation and resistance to apoptosis [82]. Shi et al. reported that reduced expression of let-7 leads to upregulation of HMGA2, promoting tumor cell growth and survival. Restoring let-7 levels could therefore suppress HMGA2 expression, offering a potential strategy to control LMS progression [29].

miR-200c plays a critical role in limiting migration and invasion. By targeting ZEB1/2, VEGFA, and TIMP2, miR-200c plays a role in the inhibition of epithelial-mesenchymal transition (EMT) and angiogenesis, which are essential processes for tumor spread and survival. The reintroduction of miR-200c into LMS cell lines has been demonstrated to diminish metastatic behavior, thereby underscoring its potential as a therapeutic target to mitigate LMS aggressiveness [34,83,84].

### 2.9. Oncogenic miRNAs in Leiomyosarcoma

Conversely, certain miRNAs act as oncogenes in LMS, promoting tumor development and metastasis.

The miR-17-92 cluster is overexpressed in uterine LMS and has been implicated in promoting cell proliferation and survival. This cluster includes several miRNAs such as miR-17 and miR-20a that target tumor suppressors and regulators of cell cycle progression. Danielson et al. found that overexpression of the miR-17-92 cluster in LMS was associated with poor differentiation and increased malignancy, suggesting its role in driving LMS pathogenesis [37]. Targeting this miRNA cluster could potentially inhibit LMS growth and improve therapeutic outcomes [37].

miR-221 has also been identified as an oncogenic miRNA in LMS, particularly in the uterine subtype. Nuovo and Schmittgen showed that miR-221 is significantly upregulated in LMS compared with benign leiomyomas [38]. This miRNA promotes tumorigenesis by targeting the cell cycle inhibitor p27Kip1, facilitating unchecked cellular proliferation [38]. The high expression levels of miR-221 in LMS make it a potential biomarker for distinguishing malignant LMS from benign smooth muscle tumors, as well as a target for therapeutic intervention [38].

Furthermore, miR-93 and miR-106b contribute to the oncogenic landscape of LMS by targeting multiple pathways involved in cell proliferation and migration. It has been demonstrated that these miRNAs facilitate tumorigenesis by downregulating pivotal regulators such as F3, IL8, CTGF, and PAI1, thereby promoting enhanced cellular growth and metastatic potential in LMS. The coordinated overexpression of miR-93 and miR-106b has been linked to increased aggressiveness and a poorer prognosis in LMS, indicating that these miRNAs may represent promising therapeutic targets for the inhibition of tumor progression in LMS [35,83].

The unique miRNA expression profiles in LMS suggest valuable potential for their application as diagnostic and prognostic markers. For example, miR-199b-5p shows notably lower expression in LMS compared with other sarcoma types like undifferentiated pleomorphic sarcoma (UPS) [39]. Guled et al. reported that lower levels of miR-199b-5p are associated with poorer prognosis and increased metastatic potential in LMS, suggesting that this miRNA could serve as a useful biomarker for patient stratification and prognosis [39].

Similarly, miR-320a is upregulated in LMS and may also have diagnostic value. Comparative studies have shown that miR-320a levels are higher in LMS than in UPS, indicating that it could help distinguish between these two histologically similar but clinically distinct sarcoma types [26]. The differential expression of miR-320a could also provide insights into the molecular mechanisms driving LMS and potentially guide therapeutic decision-making [26].

miRNA dysregulation is central to the development of LMS, affecting crucial mechanisms that drive cell proliferation, programmed cell death, and metastasis. Tumor-suppressive miRNAs like miR-152 and the let-7 family help to curb tumor growth, while oncogenic miRNAs such as the miR-17-92 cluster and miR-221 contribute to the progression and aggressiveness of the disease. These miRNAs not only deepen our understanding of the molecular landscape of LMS but also hold potential as valuable diagnostic and prognostic biomarkers. Moving forward, research should prioritize confirming the clinical utility of these miRNAs and advancing miRNA-based therapies to provide more targeted treatments and improve patient outcomes in LMS.

### 2.10. miRNAs in Synovial Sarcoma

Synovial sarcoma (SS) is a high-grade malignancy that constitutes about 5-10% of all STS [85,86,87]. It predominantly affects adolescents and young adults and is characterized by a specific chromosomal translocation between chromosomes X and 18, resulting in the formation of fusion oncogenes—SS18-SSX1, SS18-SSX2, or SS18-SSX4 [88]. These fusion proteins play a crucial role in tumorigenesis by disrupting gene regulation and chromatin remodeling. Emerging research has revealed substantial miRNA dysregulation in synovial sarcoma, playing a key role in driving tumor growth, advancement, and metastasis. Depending on their specific targets and biological context, these miRNAs can act as either oncogenes, promoting malignancy, or tumor suppressors, inhibiting cancer progression [36].

### 2.11. Oncogenic miRNAs in Synovial Sarcoma

miR-183 is one of the most extensively studied oncogenic miRNAs in synovial sarcoma. It is significantly overexpressed in synovial sarcoma cells, as well as in other cancers like RMS and colon cancer [16,26,89]. Sarver et al. demonstrated that miR-183 promotes tumor cell migration and invasion by targeting tumor suppressors such as early growth response protein 1 (EGR1) and phosphatase and tensin homolog (PTEN) [15]. That study found that the SS18-SSX fusion protein repressed EGR1 expression by directly binding to its promoter, while miR-183 further suppressed EGR1 and PTEN expression at the post-transcriptional level, enhancing oncogenic potential [15].

The miR-17-92 cluster, which includes miR-17, is also upregulated in synovial sarcoma and is induced by the SS18-SSX fusion gene [36]. This cluster acts as an oncogene by directly targeting cyclin-dependent kinase inhibitor 1 (p21, CDKN1A), which is crucial for regulating the G1 to S-phase transition in the cell cycle. Minami et al. found that overexpression of miR-17 in synovial sarcoma cells led to reduced p21 levels, promoting cell proliferation and tumor growth [90]. Conversely, blocking miR-17 resulted in increased p21 expression and reduced cell proliferation, suggesting that miR-17 plays a critical role in synovial sarcoma pathogenesis [90].

Other miRNAs, such as miR-200b and miR-375, have been found to be significantly upregulated in synovial sarcoma [91]. Hisaoka et al. identified these miRNAs among a group of 21 miRNAs that were differentially expressed in synovial sarcoma compared with Ewing’s sarcoma and normal skeletal muscle [91]. The functional analysis revealed that these miRNAs target genes were involved in cell proliferation and chromatin remodeling, contributing to the malignant phenotype of synovial sarcoma [91].

### 2.12. Tumor Suppressor miRNAs in Synovial Sarcoma

While some miRNAs act as oncogenes, others function as tumor suppressors, inhibiting tumor growth and metastasis in synovial sarcoma.

miR-143 is one such miRNA that is significantly downregulated in synovial sarcoma compared with other sarcoma subtypes, including LMS [92]. Subramanian et al. demonstrated that miR-143 targets ERK5 (also known as MAPK7), which is known to promote cell growth and proliferation in response to tyrosine kinase signaling [92]. The downregulation of miR-143 in synovial sarcoma allows unchecked ERK5 expression, contributing to increased cell proliferation and tumor growth. The reintroduction of miR-143 into synovial sarcoma cells resulted in decreased ERK5 levels, reduced proliferation, and increased apoptosis, highlighting its potential as a therapeutic target [92].

The let-7 family, including let-7e, is also downregulated in synovial sarcoma. Hisaoka et al. found that the let-7 family targets several oncogenes, including HMGA2 and SMARCA5, both of which are associated with increased cell proliferation and tumor aggressiveness [91]. Silencing let-7e and miR-99b in synovial sarcoma cells resulted in suppression of cell proliferation, suggesting that these miRNAs act as tumor suppressors by modulating the expression of genes critical for tumor progression [91].

The distinct expression profiles of miRNAs in synovial sarcoma provide valuable insights for their use as diagnostic and prognostic biomarkers. Recent study identified a panel of seven miRNAs, including miR-99a-5p, miR-146b-5p, and miR-223-3p, that were significantly upregulated in synovial sarcoma patients compared with those with other sarcomas and healthy controls [93]. This miRNA signature was able to distinguish synovial sarcoma from other sarcoma subtypes and could potentially be used for early diagnosis and monitoring of disease progression [93].

### 2.13. miRNAs in Fibrosarcoma

Fibrosarcoma is a rare and aggressive type of STS characterized by the presence of immature proliferating fibroblasts or undifferentiated anaplastic spindle cells [94]. Despite its rarity, fibrosarcoma presents significant clinical challenges due to its high rate of local recurrence and potential for metastasis. The molecular pathogenesis of fibrosarcoma involves various genetic and epigenetic alterations, including the dysregulation of miRNAs. miRNAs in fibrosarcoma play a crucial role in regulating tumor cell proliferation, migration, invasion, and metastasis, functioning either as tumor suppressors or oncogenes.

### 2.14. Oncogenic miRNAs in Fibrosarcoma

A range of miRNAs have been implicated in fibrosarcoma as key oncogenic factors, accelerating both tumor growth and metastatic dissemination via multiple mechanisms.

miR-520c and miR-373 are two miRNAs that have been shown to act as oncogenes in fibrosarcoma. Liu and Wilson demonstrated that these miRNAs upregulate the expression of matrix metalloproteinase-9 (MMP9) by directly targeting the 3′-untranslated regions (3’UTRs) of mRNAs for mechanistic target of rapamycin (mTOR) and sirtuin 1 (SIRT1), leading to their suppressed translation [42]. This suppression activates the Ras/Raf/MEK/Erk signaling pathway and nuclear factor kappa-light-chain-enhancer of activated B cells (NF-κB), enhancing MMP9 transcription and translation. The increased MMP9 levels promote extracellular matrix degradation, enhancing tumor cell migration and invasion, thereby contributing to the aggressive nature of fibrosarcoma [42].

In another study, miR-21 was identified as being upregulated in fibrosarcoma and associated with increased cellular proliferation and invasion [43]. This miRNA targets several tumor suppressor genes, including PTEN and PDCD4, both of which are involved in apoptosis and cell cycle regulation. The downregulation of these genes by miR-21 contributes to tumor growth and resistance to apoptosis, suggesting that miR-21 functions as an oncogene in fibrosarcoma [95].

### 2.15. Tumor Suppressor miRNAs in Fibrosarcoma

On the other hand, some miRNAs have been recognized as tumor suppressors in fibrosarcoma, playing a crucial role in halting tumor progression and metastasis.

The miR-29 family (miR-29a, miR-29b, miR-29c) plays a tumor-suppressive role in fibrosarcoma by targeting MMP2, a matrix metalloproteinase involved in extracellular matrix remodeling and tumor invasion [13]. The overexpression of miR-29 family members in fibrosarcoma cell lines reduced MMP2 expression, impairing cell invasion and migration [13]. Such downregulation of MMP2 is associated with decreased metastatic potential, suggesting that miR-29 could serve as a therapeutic target to reduce the invasiveness of fibrosarcoma cells.

miR-409-3p is another tumor-suppressive miRNA identified in fibrosarcoma. Weng et al. reported that miR-409-3p targets angiogenin (ANG), a potent promoter of angiogenesis and tumor growth [45]. Overexpression of miR-409-3p in fibrosarcoma cell lines led to reduced ANG expression, resulting in decreased tumor growth, vascularization, and metastasis both in vitro and in vivo. Research suggests that miR-409-3p could be leveraged as a therapeutic agent to inhibit angiogenesis and tumor progression in fibrosarcoma [45].

The varied expression patterns of miRNAs in fibrosarcoma present a valuable opportunity for their potential use as diagnostic and prognostic markers. For example, the upregulation of miR-21 has been associated with a more aggressive phenotype and poorer prognosis in fibrosarcoma patients, indicating its potential as a biomarker for disease progression and response to therapy [96].

Another important tumor-suppressive miRNA in fibrosarcoma is miR-197, which has been shown to significantly influence pathways related to cell proliferation, metastasis, and chemoresistance. In fibrosarcoma cell lines, notably HT1080, miR-197 has been observed to be downregulated, which has been demonstrated to result in enhanced tumorigenic properties. Restoration of miR-197 expression has been demonstrated to markedly diminish cell viability, impede migration, and induce apoptosis. This suppression appears to be mediated through the direct targeting of oncogenic proteins such as RAN and KIAA0101. For example, miR-197-3p negatively regulates RAN, a protein involved in cell cycle progression and proliferation, thereby promoting G2/M phase arrest and autophagy in fibrosarcoma cells [25,97]. Similarly, miR-197-5p has been demonstrated to target KIAA0101, thereby reducing metastatic behavior. This is achieved by repressing fibrosarcoma cell migration and invasion while promoting cellular senescence [98]. Furthermore, miR-197-5p has been demonstrated to augment the efficacy of doxorubicin in fibrosarcoma by reducing the expression of multidrug resistance (MDR) genes, including ABCC1 and MVP. This improves drug retention and cytotoxicity in cancer cells. This combined treatment approach demonstrates significant potential for overcoming chemoresistance in fibrosarcoma through increased apoptosis and ROS production [25].

Moreover, the downregulation of tumor-suppressive miRNAs like the miR-29 family and miR-409-3p correlates with increased tumor invasiveness and poor clinical outcomes. Restoring the levels of these miRNAs could not only serve as a therapeutic strategy but also help in stratifying patients based on their risk of metastasis and likelihood of response to specific therapies [45].

Altered miRNA expression in fibrosarcoma is central to the disease’s development, influencing vital processes such as cell proliferation, apoptosis, migration, and invasion. Certain miRNAs, like miR-520c, miR-373, and miR-21, drive tumor progression and metastasis by acting as oncogenes, while others, including the miR-29 family and miR-409-3p, serve as tumor suppressors, restraining tumor growth. Elucidating the distinct functions of these miRNAs sheds light on the molecular pathways that fuel fibrosarcoma’s aggressive behavior and highlights their value as biomarkers for diagnosis and prognosis and as potential therapeutic targets. Further studies are necessary to confirm these findings in clinical contexts and to explore miRNA-based treatment strategies for fibrosarcoma.

### 2.16. miRNAs in Angiosarcoma

Angiosarcoma is a rare and highly aggressive subtype of STS that arises from the endothelial cells lining blood vessels [99]. It accounts for less than 1% of all sarcomas and is mostly found in the skin, particularly on the scalp and face, but can also occur in deep soft tissues, the liver, heart, and breast [99,100]. Due to its aggressive nature, angiosarcoma often presents with a high rate of metastasis and poor prognosis. The pathogenesis of angiosarcoma is not well understood, but recent studies have highlighted the dysregulation of miRNAs as significant contributors to its development and progression. These miRNAs act as oncogenes or tumor suppressors, influencing various cellular processes such as proliferation, apoptosis, and angiogenesis.

### 2.17. Oncogenic miRNAs in Angiosarcoma

miR-17-92 cluster: The miR-17-92 cluster, a well-known oncogenic miRNA cluster, is significantly upregulated in angiosarcoma, especially in cases with MYC amplification. Italiano et al. identified that MYC amplification was present in three out of six primary angiosarcomas and 8 out of 12 secondary angiosarcomas [101]. This cluster, which includes miRNAs such as miR-17, miR-18a, miR-19a, miR-19b, miR-20a, and miR-92a, is known to enhance cell proliferation and survival. The overexpression of the miR-17-92 cluster in MYC-amplified angiosarcomas was associated with the downregulation of thrombospondin-1 (THBS1), a glycoprotein that inhibits angiogenesis. The suppression of THBS1 facilitates an angiogenic phenotype, contributing to the aggressive nature of angiosarcoma [101].

miR-520c-3p, miR-519a, and miR-520h: These miRNAs are also significantly upregulated in angiosarcoma [40,102]. Sarver et al. used the Sarcoma miRNA Expression Database (S-MED) to show that these miRNAs are overexpressed by more than 80-fold in angiosarcoma compared with other sarcomas [41]. Although the specific cellular functions of miR-520c-3p, miR-519a, and miR-520h in angiosarcoma have not been fully elucidated, their marked overexpression suggests a potential role in promoting tumorigenesis and metastasis [41].

### 2.18. Tumor Suppressor miRNAs in Angiosarcoma

miR-497-5p has been identified as a tumor suppressor miRNA in angiosarcoma [103,104]. Chen et al. found that miR-497-5p targets the potassium intermediate/small conductance calcium-activated channel, subfamily N, member 1 (KCa3.1), which is involved in cell proliferation and invasion [46]. The downregulation of miR-497-5p leads to increased expression of KCa3.1, enhancing tumor growth and invasion. Experimental upregulation of miR-497-5p resulted in decreased KCa3.1 expression, reduced cell proliferation, impaired cell cycle progression, and decreased invasion, suggesting that miR-497-5p could serve as a potential therapeutic target [46].

miR-378a-3p and miR-483-5p: These miRNAs are also downregulated in angiosarcoma and have been implicated in the regulation of tumor angiogenesis and growth. Reduced expression of miR-378a-3p and miR-483-5p is associated with enhanced angiogenic potential and aggressiveness of the tumor [26]. These miRNAs typically target pro-angiogenic factors and genes involved in cell survival and proliferation, and their downregulation contributes to the pro-tumorigenic environment observed in angiosarcoma [26].

The altered miRNA landscape in angiosarcoma highlights new potential for these molecules to serve as key diagnostic and prognostic indicators. The significant upregulation of the miR-17-92 cluster in MYC-amplified angiosarcomas could potentially serve as a biomarker to identify patients with a more aggressive form of the disease, aiding in the stratification of patients and the tailoring of therapeutic strategies [101].

Additionally, the downregulation of miR-497-5p suggests its potential use as a prognostic marker, as its expression inversely correlates with tumor aggressiveness and metastatic potential [103,104]. Future studies are warranted to validate these findings and explore the clinical utility of miRNA-based diagnostics and therapeutics in angiosarcoma management.

The imbalance of miRNAs in angiosarcoma significantly contributes to its progression, affecting key mechanisms such as vascular formation, cellular proliferation, and invasiveness. Pro-tumor miRNAs like the miR-17-92 cluster and miR-520c-3p drive both growth and angiogenesis, while miR-497-5p and miR-378a-3p act as tumor suppressors, hindering these malignant processes. Decoding the intricate roles of these miRNAs in angiosarcoma biology opens up new possibilities for their use in diagnostics and prognosis, as well as for therapeutic intervention. Future research should confirm these miRNAs’ clinical relevance and investigate their potential for guiding miRNA-targeted treatments that can better manage angiosarcoma in patients.

### 2.19. miRNAs in Undifferentiated Pleomorphic Sarcoma

UPS, previously known as malignant fibrous histiocytoma (MFH), varies in frequency depending on different regions of the world. In some areas, it is as common as liposarcoma and leiomyosarcoma. Additionally, UPS is considered among the most frequent soft tissue sarcomas in the extremities and in adult patients [105]. UPS is characterized by a high degree of cellular atypia and pleomorphism, lacking any specific line of differentiation, which makes it a diagnosis of exclusion [105]. Despite advances in treatment, the prognosis for UPS patients remains poor due to frequent local recurrence and metastasis. Emerging studies have revealed that miRNAs are key players in the molecular dynamics of UPS, acting as both promoters and inhibitors of tumor progression by regulating essential processes like cell proliferation, apoptosis, and metastatic spread.

### 2.20. Oncogenic miRNAs in UPS

miR-199b-5p has been found to be significantly upregulated in UPS compared with other sarcoma subtypes like LMS [26]. According to Guled et al., miR-199b-5p is involved in the regulation of genes associated with cell proliferation and differentiation [39]. The high expression levels of miR-199b-5p in UPS suggest its role in maintaining the undifferentiated state of the tumor cells and promoting tumor growth [39].

miR-1260 and miR-1274a were also found to be significantly downregulated in UPS. These miRNAs are known to target oncogenes involved in cell cycle regulation and apoptosis. The downregulation of miR-1260 and miR-1274a in UPS contributes to the loss of control over cell proliferation and survival, further promoting tumor progression [47].

### 2.21. Tumor Suppressor miRNAs in UPS

miR-152 is a notable tumor suppressor miRNA that is downregulated in UPS. This miRNA targets the receptor tyrosine kinases MET and KIT, which are crucial for cell growth and survival [33,106]. The downregulation of miR-152 leads to increased expression of MET and KIT, activating downstream signaling pathways such as PI3K/AKT, promoting cell proliferation and survival. Experimental upregulation of miR-152 in UPS cell lines was shown to reduce MET and KIT levels, inhibit the PI3K/AKT pathway, induce cell cycle arrest, and enhance apoptosis [33].

miR-320a is another miRNA that is significantly downregulated in UPS compared with LMS [40]. This miRNA targets multiple genes involved in cell proliferation and metastasis. The downregulation of miR-320a in UPS contributes to increased tumor aggressiveness and metastatic potential. The differential expression of miR-320a between UPS and LMS could also serve as a useful biomarker for distinguishing between these two sarcoma subtypes [40].

Variations in miRNA expression in UPS highlight its potential as a powerful tool for diagnosis and prognosis. For example, the significant upregulation of miR-199b-5p and the downregulation of miR-320a and miR-152 could potentially serve as biomarkers to distinguish UPS from other sarcoma subtypes and to identify patients with a more aggressive form of the disease. The differential expression of these miRNAs can also provide insights into the molecular mechanisms driving UPS and could guide therapeutic decision making.

Aberrant miRNA expression in UPS significantly impacts the molecular mechanisms driving tumorigenesis, particularly in pathways governing cell growth, death, and metastasis. Oncogenic miRNAs, including miR-199b-5p and miR-1260, fuel tumor progression and aggressiveness, while tumor-suppressing miRNAs like miR-152 and miR-320a work to counterbalance these malignant processes. Decoding the regulatory influence of these miRNAs sheds light on UPS development and offers promising opportunities for their use as biomarkers and therapeutic targets. Moving forward, research must aim to confirm the roles of these miRNAs in clinical environments and advance the creation of miRNA-based treatments to enhance patient outcomes in UPS.

### 2.22. miRNAs in Malignant Peripheral Nerve Sheath Tumor (MPNST)

MPNSTs are aggressive sarcomas arising from the peripheral nerves or cells associated with nerve sheaths, such as Schwann cells [107]. They are relatively rare and are often associated with type 1 neurofibromatosis (NF1), a genetic disorder that increases the risk of developing benign and malignant nerve sheath tumors [107]. MPNSTs are highly aggressive, with poor prognosis and a propensity for local recurrence and distant metastasis. Different research has revealed numerous miRNAs that contribute to the development of MPNST, functioning to either promote or suppress tumor activity by regulating essential processes such as cell division, cell death, and the spread of cancer cells.

### 2.23. Oncogenic miRNAs in MPNST

miR-21 is one of the most well-studied oncogenic miRNAs in MPNST. It is significantly upregulated in MPNST tissues compared with benign neurofibromas and normal nerve tissues. Itani et al. demonstrated that miR-21 targets programmed cell death protein 4 (PDCD4), a tumor suppressor involved in inducing apoptosis via a caspase cascade [44]. The overexpression of miR-21 leads to the downregulation of PDCD4, thereby reducing apoptosis and promoting cell survival and proliferation. Silencing miR-21 in MPNST cell lines has been shown to induce apoptosis and reduce cell proliferation, suggesting that miR-21 is a key driver of MPNST pathogenesis and could be a potential therapeutic target [44].

miR-10b is another oncogenic miRNA upregulated in MPNST. Chai et al. reported that miR-10b was overexpressed in primary Schwann cells isolated from neurofibromas, MPNST cell lines, and tumor tissues [108]. miR-10b targets NF1 mRNA, contributing to the dysregulation of the RAS signaling pathway, a critical driver of tumorigenesis in NF1-associated and sporadic MPNSTs. The inhibition of miR-10b in MPNST cells resulted in reduced cell proliferation, migration, and invasion, highlighting its role in promoting tumor aggressiveness and its potential as a therapeutic target [108].

miR-155 has been identified as a prominent oncogenic miRNA in MPNST, with a notable elevation in Schwann cells derived from patients with NF1. Its overexpression contributes to the acquisition of tumorigenic properties by promoting the proliferation of Schwann cells and the growth of plexiform neurofibroma (PNF), which are precursors to malignant transformation in NF1 patients. It has been postulated that miR-155 enhances proliferation by interacting with pathways involved in inflammation and immune response, thereby potentially driving MPNST progression. In vivo studies provide further support for this role, demonstrating that global inhibition of miR-155 results in a significant reduction in tumor size and improved survival in NF1 models. This highlights the potential of miR-155 as a promising therapeutic target for MPNST management [51]. Additionally, miR-27a and miR-27b are highly expressed in MPNST and contribute to its malignant behavior by directly targeting the NF1 gene, which is a key tumor suppressor. Elevated levels of miR-27a/b have been demonstrated in Schwann cells and MPNST cell lines, where they have been shown to downregulate NF1, thereby promoting proliferative and invasive behaviors that are essential to MPNST progression. Functional studies indicate that miR-27a/b also enhances angiogenesis and chemoresistance in tumor cells, making these miRNAs promising candidates for therapeutic targeting aimed at restoring NF1 levels and reducing tumor aggressiveness [52].

miR-214 is also upregulated in MPNSTs, and it is a direct transcriptional target of TWIST1, a key regulator of metastasis [48]. TWIST1 is highly expressed in MPNSTs, and the TWIST1-miR-214 axis promotes tumor progression by targeting the tumor suppressor gene PTEN, which negatively regulates the PI3K/AKT signaling pathway [48]. This signaling pathway is crucial for cell survival and proliferation, suggesting that miR-214 enhances tumor growth and resistance to apoptosis.

### 2.24. Tumor Suppressor miRNAs in MPNST

miR-34a is a significant tumor suppressor miRNA downregulated in MPNST. It is known to regulate genes involved in cell cycle progression and apoptosis. Subramanian et al. reported that p53 inactivation is common in MPNST, leading to downregulation of miR-34a [48]. The restoration of miR-34a expression in MPNST cell lines promoted apoptosis and reduced cell proliferation, indicating that miR-34a plays a critical role in maintaining cellular homeostasis and preventing tumor progression. The p53-miR-34a axis could be a promising therapeutic target in MPNST, especially given the p53 inactivation observed in these tumors [48].

miR-29c is another tumor suppressor miRNA that is downregulated in MPNST [49]. It targets matrix metalloproteinase-2 (MMP2), an enzyme involved in degrading the extracellular matrix, which facilitates tumor invasion and metastasis [49]. The experimental upregulation of miR-29c in MPNST cells resulted in reduced MMP2 activity, decreased invasion, and impaired metastatic potential, highlighting its potential as a therapeutic agent to control MPNST aggressiveness [49].

miR-204 is downregulated in MPNST and targets oncogenes such as Ras and HMGA2 [50]. Gong et al. found that restoring miR-204 expression in MPNST cell lines reduced cell proliferation and invasion, suggesting its role as a tumor suppressor in these tumors [50]. miR-204 may also regulate pathways critical for cell differentiation, further highlighting its potential utility in therapeutic strategies aimed at controlling tumor growth and progression in MPNST [50].

The miRNA expression profiles observed in MPNST present considerable opportunities for diagnostic and prognostic advancements. An example of this is the increased expression of miR-21, miR-10b, and miR-214 in MPNST tissues, which could help differentiate malignant tumors from benign neurofibromas, facilitating early diagnosis and more precise treatment planning. Additionally, the downregulation of tumor-suppressive miRNAs such as miR-34a and miR-29c correlates with increased tumor aggressiveness and poor clinical outcomes, suggesting their potential as prognostic markers. While miRNAs like miR-21, miR-10b, and miR-214 drive tumor growth and progression, others such as miR-34a, miR-29c, and miR-204 act as tumor suppressors, countering these effects. A deeper understanding of the miRNA landscape in MPNST not only sheds light on the mechanisms behind this cancer but also highlights their potential as biomarkers and therapeutic targets. Further studies should prioritize validating these insights for clinical applications and developing miRNA-based treatments for MPNST.

## 3. Conclusions and Future Prospective 

The intricate role of miRNA dysregulation in the pathogenesis of STSs has unveiled new molecular insights into tumor biology. Across various sarcoma subtypes, including RMS, liposarcoma, leiomyosarcoma, synovial sarcoma, fibrosarcoma, angiosarcoma, UPS, and MPNST, miRNAs serve as both oncogenes and tumor suppressors, influencing key processes such as cell proliferation, differentiation, apoptosis, migration, and metastasis. Their dual roles underscore the complexity of sarcoma progression, with miRNAs not only driving malignancy but also offering pathways for potential therapeutic intervention. As the molecular landscape of sarcomas becomes clearer, the identification of miRNAs that are consistently dysregulated across different subtypes presents an opportunity for the development of miRNA-based diagnostics, prognostics, and therapeutic strategies. For instance, miRNAs like miR-21, miR-143, and the let-7 family have shown significant potential across multiple sarcoma types, functioning as both biomarkers for disease progression and targets for therapy.

Future research should aim at translating these molecular findings into clinical applications. Priorities include validating miRNA-based biomarkers in large-scale clinical trials, optimizing miRNA delivery systems for targeted therapies, and exploring combination treatments where miRNA modulation can enhance existing chemotherapy or immunotherapy regimens. Given the aggressive nature and poor prognosis associated with many sarcomas, miRNA-based approaches could significantly improve patient outcomes, offering more personalized and effective treatment options.

## Figures and Tables

**Table 1:** Summary of soft tissue sarcoma miRNAs.

miRNA	Role	Tumor
miR-1	Promotes muscle differentiation [11]	RMS
miR-206	Promotes muscle differentiation [12]	RMS
miR-133a/b	Regulates proliferation/differentiation [11]	RMS
miR-29 family	Tumor suppressor [13,14]	RMS/Fibrosarcoma
miR-183	Promotes migration/invasion [15,16]	RMS/Synovial Sarcoma
miR-9	Promotes metastasis [17]	RMS
miR-214	Inhibits tumor growth [18]	RMS
miR-203	Tumor suppressor [19]	RMS
miR-450b-5p	Growth and differentiation [20]	RMS
miR-22	Inhibits tumor growth [21]	RMS
miR-28-3p	Tumor suppressor [22]	RMS
miR-27a	Tumor suppressor [23]	RMS
miR-181a/212	Tumor suppressor [24]	RMS
miR-197	Tumor suppressor [25]	Fibrosarcoma
miR-378a-3p	Modulates apoptosis/migration [21,26]	RMS/Angiosarcoma
miR-143	Tumor suppressor [27]	Liposarcoma
miR-145	Tumor suppressor [28]	Liposarcoma
miR-451	Inhibits proliferation [28]	Liposarcoma/UPS
Let-7 family	Tumor suppressor [29]	Liposarcoma/Leiomyosarcoma
miR-155	Oncogene [30]	Liposarcoma
miR-26a-2	Oncogene [31]	Liposarcoma
miR-486	Tumor suppressor [32]	Liposarcoma
miR-152	Tumor suppressor [27,33]	Leiomyosarcoma/UPS
miR-200c	Tumor suppressor [34]	LMS
miR-93	Oncogene [35]	LMS
miR-106b	Oncogene [35]	LMS
miR-17-92	Oncogene [36,37]	Leiomyosarcoma/Ewing’s sarcoma
miR-221	Oncogene [38]	Leiomyosarcoma
miR-199b-5p	Tumor suppressor [39]	UPS
miR-320a	Tumor suppressor [26,40]	Leiomyosarcoma/UPS
miR-520c	Oncogene [41,42]	Fibrosarcoma/Angiosarcoma
miR-373	Oncogene [42]	Fibrosarcoma
miR-21	Oncogene [43,44]	Fibrosarcoma/MPNST
miR-409-3p	Tumor suppressor [45]	Fibrosarcoma
miR-497-5p	Tumor suppressor [46]	Angiosarcoma
miR-483-5p	Tumor suppressor [26]	Angiosarcoma
miR-1260	Tumor suppressor [47]	UPS
miR-1274a	Tumor suppressor [47]	UPS
miR-34a	Tumor suppressor [48]	UPS
miR-29c	Tumor suppressor [49]	MPNST
miR-204	Tumor suppressor [50]	MPNST
miR-155	Oncogene [51]	MPNST
miR-27a/b	Oncogene [52]	MPNST

RMS = rhabdomyosarcoma; UPS = undifferentiated pleomorphic sarcoma; MPNST = malignant peripheral nerve sheath tumors.

## Data Availability

All data relevant to the study are included in the article.
